# Inverse folding based pre-training for the reliable identification of intrinsic transcription terminators

**DOI:** 10.1371/journal.pcbi.1010240

**Published:** 2022-07-07

**Authors:** Vivian B. Brandenburg, Franz Narberhaus, Axel Mosig

**Affiliations:** 1 Ruhr-University Bochum, Faculty of Biology and Biotechnology, Microbial Biology, Bochum, Germany; 2 Ruhr-University Bochum, Faculty of Biology and Biotechnology, Bioinformatics Group, Bochum, Germany; 3 Ruhr-University Bochum, Center for Protein Diagnostics, Bochum, Germany; University of Missouri, UNITED STATES

## Abstract

It is well-established that neural networks can predict or identify structural motifs of non-coding RNAs (ncRNAs). Yet, the neural network based identification of RNA structural motifs is limited by the availability of training data that are often insufficient for learning features of specific ncRNA families or structural motifs. Aiming to reliably identify intrinsic transcription terminators in bacteria, we introduce a novel pre-training approach that uses inverse folding to generate training data for predicting or identifying a specific family or structural motif of ncRNA. We assess the ability of neural networks to identify secondary structure by systematic *in silico* mutagenesis experiments. In a study to identify intrinsic transcription terminators as functionally well-understood RNA structural motifs, our inverse folding based pre-training approach significantly boosts the performance of neural network topologies, which outperform previous approaches to identify intrinsic transcription terminators. Inverse-folding based pre-training provides a simple, yet highly effective way to integrate the well-established thermodynamic energy model into deep neural networks for identifying ncRNA families or motifs. The pre-training technique is broadly applicable to a range of network topologies as well as different types of ncRNA families and motifs.

## Author summary

Intrinsic transcriptional terminators are essential regulators in determining the 3’ end of transcripts in bacteria. The underlying mechanism involves RNA secondary structure, where nucleotides fold into a specific hairpin motif. Identifying terminator sequences in bacterial genomes has conventionally been approached with well-established energy models for structural motifs. However, the folding mechanism of transcription terminators is understood only partially, limiting the success of energy-model based identification.

Neural networks have been proposed to overcome these limitations. However, their adoption for predicting and identifying RNA secondary structure has been a double edged sword: Neural networks promise to learn features that are not represented by the energy models, while they are black boxes that lack explicit modeling assumptions and may fail to account for features that are well understandable based on decades-old energy models.

Here, we introduce a pre-training approach for neural networks that uses energy-model based inverse folding of structural motifs. As we demonstrate, this approach “brings back the energy model” to identify transcriptional terminators and overcomes the limitations of previous energy-model based predictions. Our approach works for diverse types of neural networks, and is suitable for the identification of structural motifs of many other RNA molecules beyond transcriptional terminators.

This is a *PLOS Computational Biology* Methods paper.

## Introduction

The structure of non-coding RNAs (ncRNAs) plays a key role in various cellular mechanisms [1], and a thorough understanding of their structural properties is key to deciphering these mechanisms. Since probing of RNA structure experimentally is laborious, computationally predicting secondary structure from sequence often serves as starting point to investigate ncRNA secondary structure. For dealing with RNA structure computationally, numerous tools have emerged over the past decades, ranging from energy-model based folding algorithms [2, 3] to statistical models that capture ncRNA evolution at the level of sequence and structure, including profile hidden Markov models [4], covariance models [5] or heuristic approaches [6, 7]. All these now well-established approaches are founded on specific biophysical or statistical models that capture explicit assumptions about ncRNA sequence and structure.

With the advent of deep neural networks in computational biology, it has become evident that they often outperform conventional approaches in prediction or identification tasks, as prominently demonstrated in the prediction of protein structure [8], functional assignment of DNA [9], or in microscopic image analysis [10]. To no surprise, deep neural networks have been employed successfully to predict RNA secondary structure [11, 12]. While success is often limited to specific structures represented in the training data, the recent work by Sato et al. [13] achieves remarkable success with a hybrid approach that integrates the Mathews-Turner energy model into a deep neural network.

It is an inherent property of most deep neural networks that they lack explicitly stated modeling assumptions, so that they are commonly considered as black boxes whose output is opaque and lacks causal explanation [14]. This model-free approach has obvious advantages in the context of non-coding RNA, since neural networks can potentially infer spurious combinations of sequence or structure motifs or hidden correlations between those from the training data, which may be difficult or impossible to incorporate in an explicit model. One way to look at neural networks is that they possess an explicitly broad and only loosely defined inductive bias as the only methodological modeling constraint [15], and thus shift the task of modeling to the training data. If the training data contain variances that represent modeling assumptions, it is assumed that these variances will be learnable by the largely unconstrained inductive bias of the neural network. Our approach follows this line of reasoning in the context of non-coding RNA. Here, an obvious problem is the limited amount of training data for specific families or motifs of secondary structure. We tackle this problem by a model-based approach that generates training data through inverse folding of a given RNA secondary structure. The idea is to obtain a deep learning model that has explicitly learned secondary structure through inverse folding, whilst maintaining the unconstrained flexibility to learn unknown and implicit variances beyond secondary structure subsequent to pre-training.

A key question when using deep learning in the context of ncRNA is whether and, if so, how far trained models have learned representations of secondary structure. To address this, we perform systematic *in silico* mutagenesis experiments that unveil whether a neural network recognizes secondary structure elements.

We establish our *in silico* mutagenesis approach in the context of identifying intrinsic transcription terminators in bacteria. These RNA elements can be found at the 3’-end of RNAs, where they initiate the termination of transcription elongation. In distinction from other termination types, which involve proteins that either cause damaging (e.g. Mfd) or dissolving (e.g. Rho) of the elongation complex, intrinsic terminators are also known as Rho-independent terminators.

### Background

In recent years, numerous deep learning approaches have been proposed to predict RNA secondary structures with or without pseudoknots, utilizing a diversity of input encodings, output formats and network architectures.

#### Input formats

**Fig 1:**
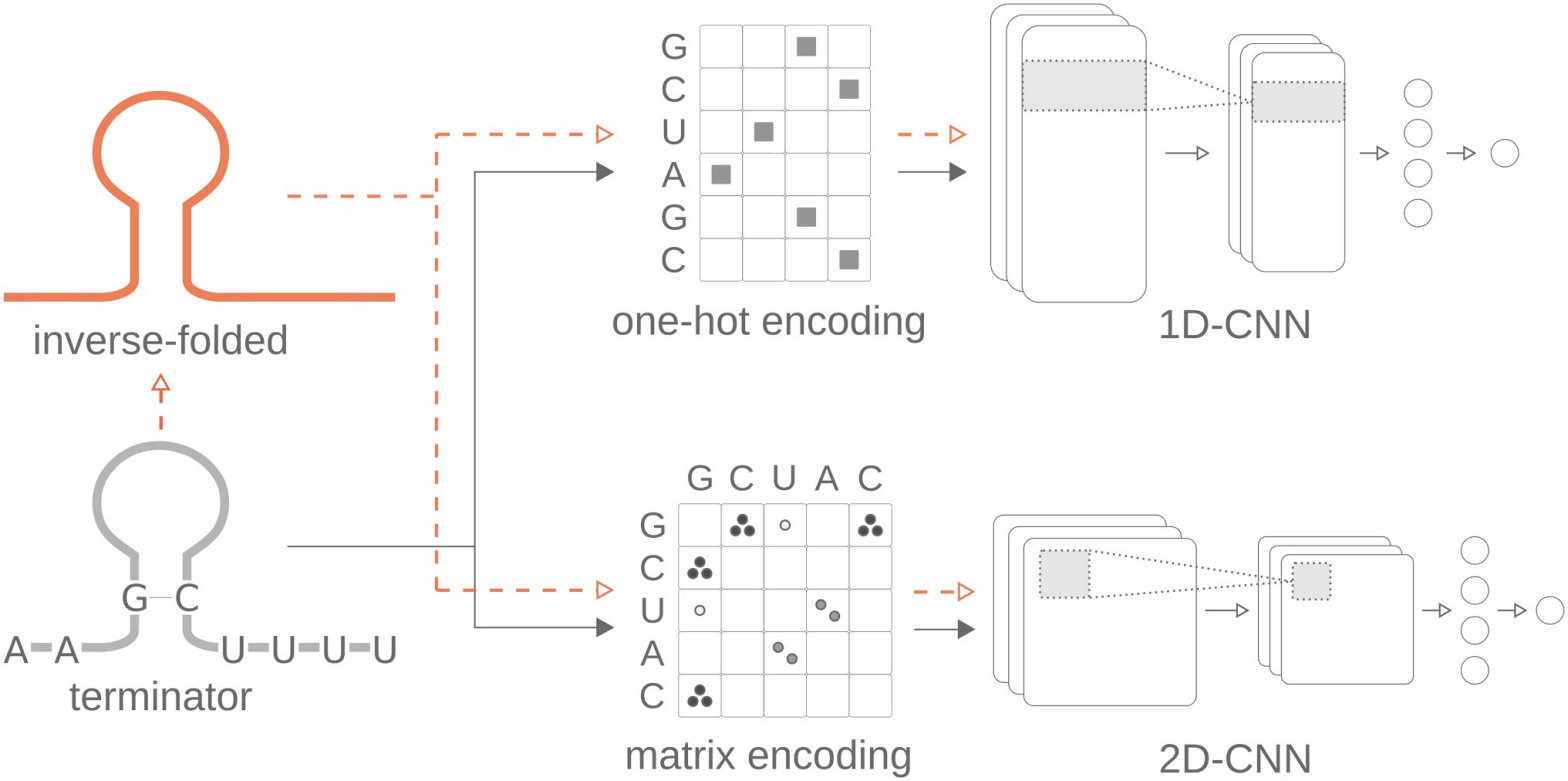
Model architectures and training strategy. The model input is formed by terminator sequences. In the pre-trained model, the models are first trained with inverse-folding based data before training with terminator sequences. These pre-training data feature the structure of terminators, but not their specific sequence properties. The input data are either one-hot encoded and fed into a 1D-CNN, or matrix encoded and then passed into a 2D-CNN. Both CNN architectures are followed by a fully connected layer and a single output neuron.

Arguably the most common input encoding for nucleotide sequences is *one-hot encoding* of the input sequence, in which each sequence position is represented by four input neurons, one for each of the four nucleotides, so that a sequence of length *L* is represented as a binary *L* × 4 matrix [12, 16–19] as displayed in Fig 1 (top). This input format has been combined with different additional information, including position embedding [16, 17], base frequency [12], and partition function [12, 20]. The other common input format transforms the sequence of length *L* into an *L* × *L* matrix [21, 22] which scores potential complementary base pairings in the sequence as displayed in Fig 1 (bottom). In a recent variant, this matrix representation was extended by encoding alternative pairings in additional channels [11, 23].

#### Network architectures

Various deep learning architectures have been used for RNA structure prediction. The smaller fraction of these utilizes long short-term memory cells (LSTMs) [12, 18, 24]. Although LSTMs are conceptually predestined for processing sequential data [25], most approaches dealing with ncRNAs utilize different variants of convolutional neural networks (CNNs) [16, 17, 21], including CNN variants derived from GoogLeNet [22] and U-Net [11]. References [16] and [17] additionally introduced the idea of enhancing important sequence features via an attention mechanism [26]. [13] combined different network architectures by stacking a CNN and LSTM layers. [19] combined ResNet blocks [27] and a 2D-bidirectional LSTM layer.

#### Post processing

The output of most deep learning models for RNA structure is a matrix representing base pair probabilities. Some authors [12, 21, 22, 24] obtain probabilities for parentheses strings from this matrix, while other authors produce base pairing probabilities for each possible base pair in an *L* × *L* matrix [11, 16, 19, 23]. In both cases, the model output may involve inconsistent base pairing patterns, so that most proposed methods include a post-processing step which maximizes the number of base pairs [24] or the probability sum [21–23] in the final output structure. In contrast to other methods, [13] predict folding scores for helix stacking, helix opening, helix closing and unpaired regions rather than pairing probabilities, and combine these with energy parameters [28]and Zuker-style dynamic programming [3].

#### Transfer learning

One common issue in deep learning is the quantitative lack of labeled data with sufficient quality. To address this problem, Singh et al. [19] introduced a transfer learning [29] approach, based on a first round of training on more than 10,000 sequences from the bpRNA [30] database with automatically generated secondary structures. The resulting model is transfer learned on a very small data set of less then 250 high-resolution RNA structures. In a further study, Singh et al. [20] combine this approach with *blastn* [31] based homology search and the covariance based secondary structure models implemented in *infernal* [32]. The neural network integrates the one-hot encoded sequence along with base pair probabilities predicting from a partition function [33]. Additionally, a direct-coupling-analysis was performed, whose output also represents an input feature for the CNN.

### Intrinsic transcription terminators

Transcription terminators are located at the 3’-end of RNA transcripts, as hindmost RNA element upstream of the transcription termination site. They ensure that the transcription process is terminated at defined 3’ends of transcripts. This prevents overflowing and incorrect transcription of adjacent genes, as well as mutual interference of transcription machinery [34]. Thus terminators are one of the basic elements for the orderly flow of regulatory processes. The forced dissociation of the transcription complex also enables the recycling of the elements involved [35].

**Fig 2:**
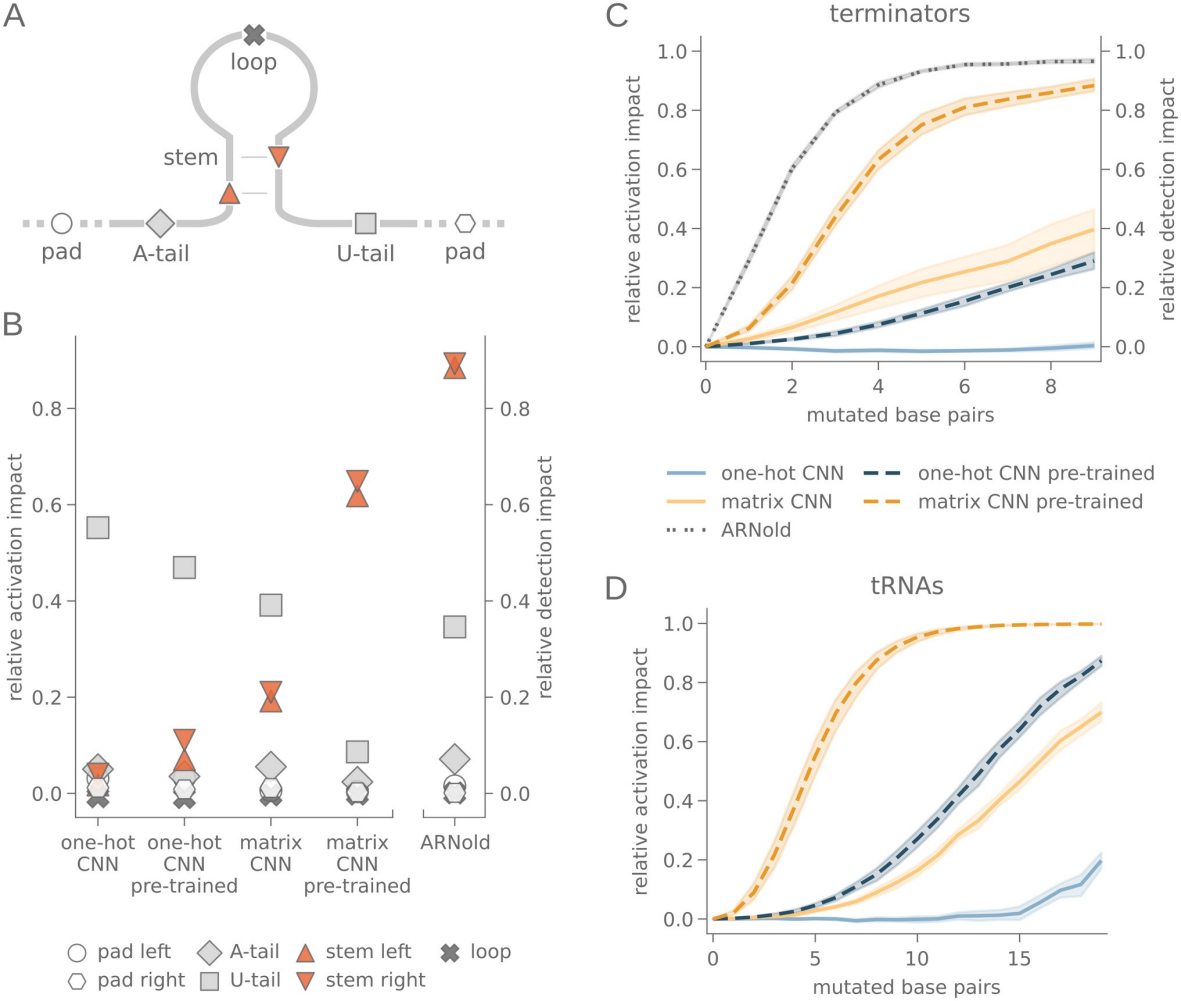
Impact of sequence and structure on terminator and tRNA recognition. (A) Intrinsic terminators comprise five sections: The hairpin structure in the center consists of a stem and a loop, framed by an A-rich zone (A-tail) on the 5’-end and a longer U-rich zone (U-tail) on the 3’-end. The terminator data used in this study additionally contain adjacent genomic sequences of the terminator (*left pad* and *right pad*). (B) Impact of terminator sections as relative activation impact on CNN models (left) and relative detection impact on ARNold (right). Random mutations were introduced in each of the 7 sections of the transcription terminators. The relative activation impact on the models is calculated from the difference between the model output corresponding to the original sequences and sequences with random nucleotide mutations in half of all nucleotides per section. The relative detection impact for ARNold is calculated for the same mutated sequences, and is estimated by averaging over binary outputs across the mutation data set. (C) Impact of the base pairings in the stem of terminators for a growing number of mutated base pairs as relative activation impact on CNN models and relative detection impact on ARNold. The relative activation impact is calculated from the difference between the model output corresponding to mutations which retain or disrupt the pairing state in the stem structure. The relative detection impact for ARNold is calculated for the same mutated sequences, and is estimated by averaging over binary outputs across the mutation data set. (D) Relative activation impact of the base pairings in the stems of tRNAs on CNN models, for a growing number of mutated base pairs. The relative activation impact is calculated from the difference between the model output corresponding to mutations which retain or disrupt the pairing state in the stem structure. (B), (C): For *k* = 1, …, 10 and *n* ∈ {93, 84, 102, 91, 94, 92, 93, 113, 99, 92} (D): For *k* = 1, …, 10 and *n* ∈ {198, 203, 194, 202, 201, 201, 199, 201, 194, 201}.

The sequence of intrinsic terminators can be divided into several sections (Fig 2A): the center of the sequence is formed by a GC-rich hairpin. From this, an A-rich region (A-tail) stretches in 5’-direction, and a U-rich region (U-tail) can be found at the 3’-end [36] which acts as a pausing site for the RNA polymerase [37]. This pausing, enhanced by additional elements [38], temporarily suspends the change of thermodynamic parameters by constant elongation of the transcript and allows the stable hairpin to form [39]. The hairpin then extends, hijacking about 3 bp from the RNA:DNA hybrid, which destabilizes the elongation complex and eventually initiates the dissociation of the complex [40]. Nonetheless, the exact mechanistic details are still a subject of discussion [41, 42].

The importance of defined 3’-ends for the regulatory processes of transcription is widely acknowledged. Their transcriptome-wide characterization, however, has so far lagged behind that of 5’-ends. In the past decade, RNA-Seq-based methods have been developed further, which enabled the transcriptome-wide investigation of transcription start sites. These include differential RNA-Seq [43], tagRNA-Seq [44] and Cappable-seq [45]. More recently, an analogous method was developed for the targeted analysis of transcription endpoints: Term-Seq is a high-throughput sequencing approach, aiming for transcriptome-wide discovery of transcription termination sites in bacteria [46], and has been further developed to allow for direct quantification of termination efficiency [47].

Simultaneous 5’ and 3’ end sequencing (SEnd-seq) is a different approach to identify transcription termination ends, along with their associated start sites [48]. The key step in this approach is the circularization of cDNA, where 5’- and a biotin-labeled 3’-ends are ligated. After shearing of the cDNA-ring, the biotin-labeled pieces can be isolated, sequenced and used to map transcription start sites and termination sites on a nucleotide-level resolution. By identifying transcription termination sites, Term-Seq as well as SEnd-seq can indicate the position of transcription terminators.

#### Predicting intrinsic terminators

The correct annotation of intrinsic transcription terminators is an important part of the deciphering of transcription processes and their underlying rules and mechanisms. As their experimental identification is challenging, various attempts have been made to solve this problem [49–54]. To date, most automated tools for intrinsic terminator detection are based on a combination of stem stability estimation and motif finding [55–57]. Since systematic and experimentally validated annotations of terminators are only available in *Escherichia coli* and *Bacillus subtilis*, our study relies on data from these two species. Although there are remarkable similarities across the species studied to date, differences can be found as well: While the hairpin structure in *B. subtilis* contains more base pairs and is more stable, the U-stretch has a slightly larger U-content in *E. coli* [58]. The terminator efficiency of individual terminators is not necessarily transferable to other species [59]. Yet, the basic characteristics of the terminators are similar among the terminators of these two well-studied species [58, 60], and U-tail, as well as hairpin, have been demonstrated to be universal elements for functionality in bacteria [59].

## Materials and methods

### Training data for transcription terminators

Sequences of rho-independent transcription terminators from two experimental studies about *E. coli* [60] and *B. subtilis* [58] were used to train, test and validate different deep learning models. The terminator sequences were first filtered for sequence length. Terminators with a length of more than 75 nt were discarded, leaving 316 sequences from *E. coli* and 859 sequences from *B. subtilis*. Shorter sequences were padded to a length of 75 nt using the surrounding genomic sequences. Genomic sequences from random non-terminator regions from both organisms were used as negative set. The negative set was chosen with the same balance of genome origin as well as strand orientation as the terminator data. While the amount of available positive training data is limited to experimentally validated terminators, the possible negative training data are only limited by the genome size of the two species included in this study. To increase the number of data points for model training, we used threefold more negative training data than terminators. The final data set included 1175 terminator and 3525 non-terminator sequences.

### Training data for tRNAs

The tRNA sequences used for training and testing of the models were gathered from the tRNA-DB [61]. All available tRNA sequences from Gamma-Proteobacteria were included. Duplicated sequences as well as species without available genome assemblies were eliminated, leaving 1380 tRNA sequences from 48 species in the positive set. The sequences were padded to a length of 95 nt by adding the adjacent DNA up- and downstream of the tRNA. Additionally, 3906 random non-tRNA sequences were extracted from the 48 genomes, keeping the ratio of positive and negative training samples roughly the same as in the terminators data set.

### Deep learning approaches

We compare two different network topologies. First, a one-hot-encoding based CNN, henceforth referred to as *one-hot CNN* and secondly a matrix-encoding based CNN, referred to as *matrix CNN* throughout the rest of the manuscript. Each of the two topologies is assessed with and without a newly proposed pre-training approach. As a third topology, a state-of-the-art long-short-term memory recurrent neural network topology has been examined. Due to its comparatively weak performance, the topology along with validation are presented in S1 Fig.

The convolutional layer of the one-hot CNN comprises of 30 filters, with a kernel size of 10 and an rectified linear activation function. The next layer is formed by a Max-Pooling layer with a pool-size of 5 and a dropout rate of 0.2. This is followed by two fully connected layers, a first dense layer with 360 nodes, which uses a rectified linear activation function, and a second fully connected layer with 30 units and sigmoid activation function. The output layer is formed by a single output neuron. Adamax [62] was used as optimizer, in line with previous work [19]. The one-hot encoding layer encodes each nucleotide of an input sequence as a a bit-wise vector of length 4 with one high and three low bits. The encoded sequence is represented by an *L* × 4 matrix, with *L* being the sequence length.

The matrix CNN uses an *L* × *L* matrix as input, in which the self-pairing potential within the input sequence is described. To reflect the varying stability of the two Watson-Crick base pairs and the so-called wobble base pairs, G–C, A–U and G–U pairings are weighted with 1, 0.66 and 0.33, respectively. Nucleotide pairs not forming one of the three base pairs are represented as 0. For the sake of comparability with the one-hot CNN, all other parameters of the matrix CNN architecture are the same as in the one-hot CNN. The output layer of all models was formed by a single output neuron, which binary discriminates between a terminator and a non-terminator input.

### Cross validation

**Fig 3:**
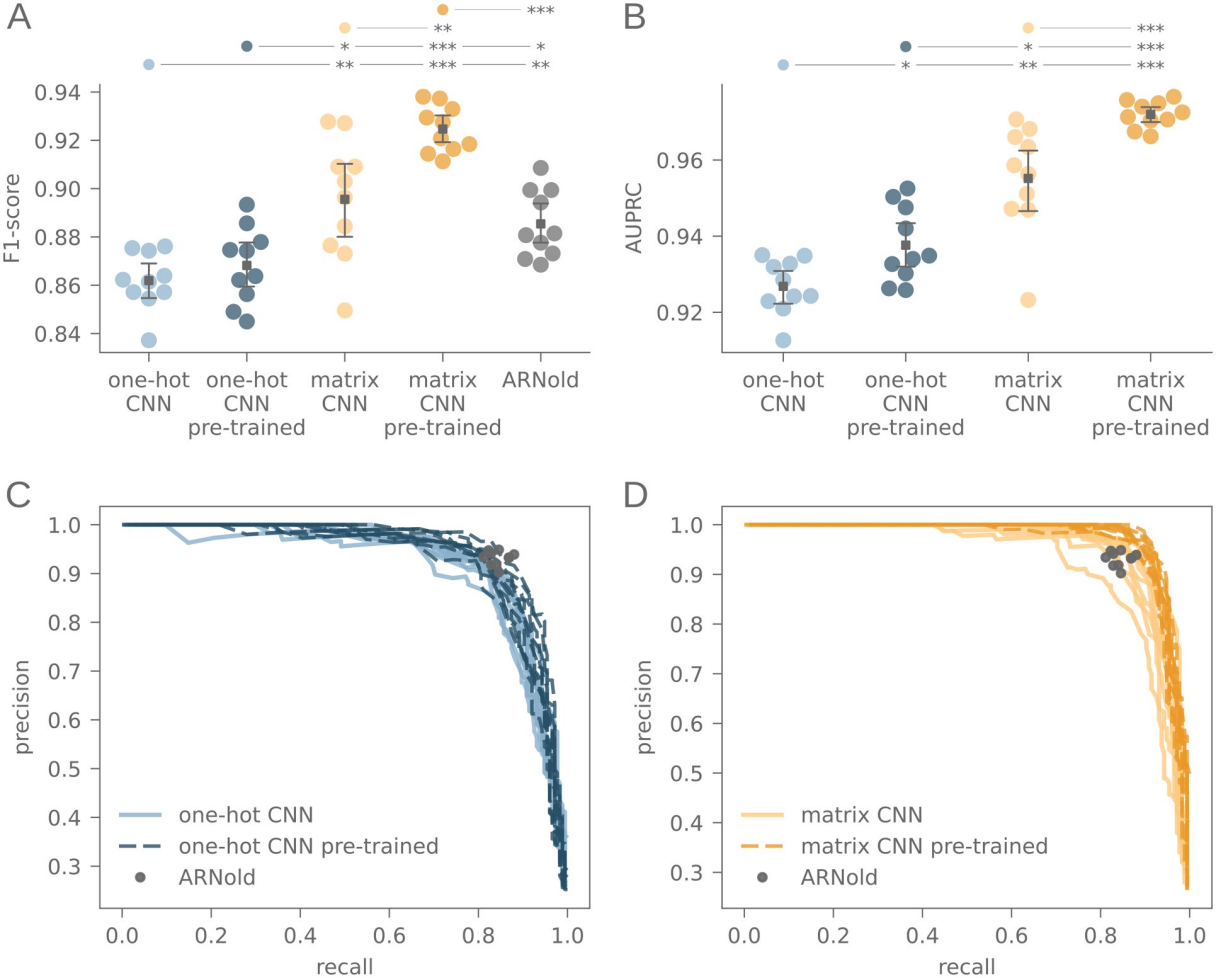
Performance comparisons. F1-score (A), area under precision-recall curve (B) and precision-recall curve (C, D) of one-hot CNN and matrix CNN with and without pre-training. The performance of ARNold on the same validation data is indicated in grey. The p-value of the Wilcoxon rank-sum test between each model *x* and *y* is indicated as coloured dot above model *x*, and as asterisks above model *y*, with *: *p* ≤ 0.05, **: *p* ≤ 0.005, ***: *p* ≤ 0.001.

For training, testing and validation, the data were randomly split into 0.70%, 0.15% and 0.15%, respectively. Monte Carlo cross-validation was used, for which the random split was carried out ten times. Each of the ten resulting data sets was used to train, test and validate all models used in this study. Throughout the training, the accuracy of the training data was used to assess model performance. The training was stopped when the accuracy of the test set did not improve further. Recall, specificity and F1-score of all models was determined on the corresponding validation set, and the area under precision-recall curve (AUPRC) was calculated for all models. The differences between the model types in F1-score as well as the AUPRC were tested with the Wilcoxon rank-sum test. The p-values of this test are indicated by asterisks in the corresponding Fig 3. Exact values are additionally stated in S2A and S2B Fig.

### Inverse-folding based pre-training

To gain training data with terminator-like secondary structures, we used the structure of *B. subtilis* terminators, as previously published by [58], and generated structure-equivalent sequences. As surveyed in [63], several algorithms have been proposed to determine sequences that fold into a given structure. We chose the well-established RNAinverse [64] for the inverse folding, which is implemented in the *RNAlib* module [65]. This process yields our pre-training set comprising 3623 terminator-shaped RNAs and 3623 random sequences of the same length. The inverse-folding generated sequences have a less pronounced GC-bias than the original transcription terminators (S3 Fig), indicating that no further sequence-bias is introduced through pre-training. 85% of the inverse-folding based data were used for the pre-training, and 15% were used to determine the early stopping point of the pre-training. 5905 random sequences with tRNA-like structures were generated accordingly, with structures deriving from the entire training set of tRNAs. The negative set for pre-training of tRNA structures was formed by 5905 random sequences of the same length. The inverse-folding data based on tRNA structures were split into a set for pre-training (80%) and a set for determining early stopping (20%).

### Validation on SEnd-seq data

To confirm the results, we cross-validated the performance of all trained models on a transcriptome-wide detection of transcription boundaries, using the SEnd-seq data set from Ju *et al*. [48]. The scan for terminators was limited to the transcribed regions, as published in the same study. To ensure that entire terminator sequences are included in the scan, each transcript was elongated with 150 nt on each end.

The prepared transcriptome was scanned with a sliding window with a step size of 3 nt. Neighbouring hits with a model output above 0.5 were fused to one hit. For each hit, the central nucleotide position and the maximum model output were used to calculate precision and recall. An additional search for terminators in the transcriptome was performed with ARNold, again using the central nucleotide as reference position for the ARNold hit.

For each trained model as well as ARNold, predicted terminators with a distance of at most 10, 15, 35, 50, 100, 150 and 250 nt to the next SEnd-seq hit were counted as true positives, and as true negatives otherwise. SEnd-seq hits with no predicted terminators were considered false negatives.

## Results

### Identifying terminators

The one-hot CNN and the matrix CNN were trained with terminator sequences from *B. subtilis* and *E. coli*, and were evaluated both with and without inverse pre-training. As a first indicator, whether the models successfully learned to identify transcription terminators, precision, recall, F1-score and AUPRC of the trained models were obtained on the validation data sets (Fig 3).

The AUPRC as well as the F1-scores of the matrix CNN are significantly higher than of the one-hot CNN, and higher when the models are pre-trained for all input types. The F1-score shows that the matrix CNNs performs similarly to ARNold, and the pre-trained matrix CNN outperforms all other models.

### Mutation experiments unravel model attention

After observing a clear performance improvement due to pre-training, the question of model interpretation arises, i.e., what sequence or structural features the model has learned during pre-training.

As a way to tackle this question, we introduce a systematic scheme for mutating terminator sequences and their secondary structure. This allows to observe the effect on model output, expecting that the model output will be most affected by mutations of important features. Conversely, changes of sequence or structural features which are not important should not result in any changes of the model output.

As displayed in Fig 2A, intrinsic transcription terminators are structurally divided into five parts through the structural main features of a GC-rich helical enclosing a hairpin loop, framed by A-residues and U-residues in the tail regions. To assess the identification of these structural components, we introduced two types of *in silico* mutagenesis experiments, referred to as *section-mutations* and *structure-mutations*. The data set for these experiments was established using all *B. subtilis* terminators with known secondary structure from each validation set. This includes between 84 and 113 sequences for each model. More specifically, 84, 91, 92, 92, 93, 93, 94, 99, 102 and 113 sequences were used for the mutation experiments for the 10 data sets.

In the section-mutation experiment (Fig 2B), we tested which of the sections of a terminator sequence (pad sequence, A-tail, stem, loop, U-tail) had an impact on the terminator recognition. For each of the 7 sections, half of all nucleotides were randomly mutated. This was repeated 15 times for each sequence in all validation sets. The relative activation impact of the section on the model was calculated for each section in every validation set as $1-({\bar{x}}_{S}/{\bar{x}}_{0})$, with ${\bar{x}}_{0}$ and ${\bar{x}}_{S}$ being the averaged model output, corresponding to the original sequences and the mutated sequences, respectively. The relative activation impact of each section on each model is displayed in S4 Fig.

As the section mutation experiments displayed in Fig 2B clearly show, pre-training strongly shifts attention towards the structured hairpin regions. This attention-shift leaves open whether the model learned RNA structure or rather a hidden sequence motif. To further investigate the impact of the stem stability on the model, we additionally introduced structure-mutations of the mutation validation set (Fig 2C). An increasing number of base pairs was randomly picked from the stem region. Both nucleotides involved in the pairing were mutated, following rules which either retained or disrupted the base pairing. This was repeated 15 times per mutation type for each sequence in each validation set. The model output for each mutation type is shown in S5 Fig. The relative activation impact of the stem stability on the model was calculated as $1-({\bar{x}}_{d}/{\bar{x}}_{r})$, with ${\bar{x}}_{r}$ and ${\bar{x}}_{d}$ denoting the averaged model output, corresponding to mutations retaining and disrupting the stem structure.

The section-mutation as well as structure-mutation sequences of all validation data were further used as input for ARNold (Fig 2B and 2C). Unlike neural networks, the output of ARNold is not a real number, but rather the number of hits detected in the tested sequence. To be able to compare both methodological approaches anyway, the rate of sequences with at least one terminator detected by ARNold was determined and used as basis for calculating the relative detection impact of the section-mutation and the structure-mutation experiments. Accordingly, the output of ARNold is not directly comparable to neural network output.

### Results of mutation studies

In the one-hot CNN without pre-training, the U-tail receives the highest attention in the section-mutation (Fig 2B), exceeding the attention of all other sections. Consistent with this observation, the destabilization of the stem has no impact on the model output (Fig 2C). Inverse folding based pre-training affects the attention pattern of the one-hot CNN substantially. This matches the expectation that inverse pre-training reduces prominence of the U-tail, while strengthening the identification of RNA structure. The influence of the U-tail is lowered, and in particular the stem region gains influence in mutation experiments (Fig 2B and 2C).

For the matrix CNN, the impact of the structured stem is considerably higher than for the one-hot CNN, and the influence of the U-tail is weakened (Fig 2B). Even without inverse pre-training, the matrix encoding clearly strengthens the identification of structuring elements. This matches expectations since the matrix encoding accounts for all possible self pairing structures within the RNA molecule.

Attention to structural features is further enhanced when combining the matrix CNN with inverse pre-training. In fact, the two stem sites can be identified as the part which has by far the largest influence on model output, nearly matching the impact pattern of ARNold (Fig 2B). The reason for this effect is apparently not, or at least not exclusively, an increased recognition of a sequence motif in the area. The destruction of the stem stability also has a drastic influence. This can be seen from the fact that the model output drops drastically with the destruction of the base pairings (Fig 2C).

In order to assess whether the observed effects of inverse pre-training and matrix encoding on the identification of stem stability extends to more complex structures, we repeated the training of all models with tRNA sequences, and repeated the introduction of retaining and destabilizing mutations in the stems of tRNAs (Fig 2D and S6 Fig). The results were similar to the findings from mutation experiments with terminators: The matrix CNN reacted stronger to destabilizing mutations compared to the one-hot CNN. The same effect was observed when the model was pre-trained with inversely generated sequences. This confirms that both methods shift the focus of the model from the nucleotide sequence to the secondary structure.

In summary, we observe that inverse pre-training consistently and significantly affects attention towards secondary structure, with matrix encoded input starting at a higher baseline than one-hot encoded input.

### Transcriptome annotation

In order to assess whether neural networks are suitable for detecting RNA elements on a transcriptome-wide scale, we tested our trained models on the transcription termination sites of *E. coli* using the SEnd-seq-based results from [48].

**Fig 4:**
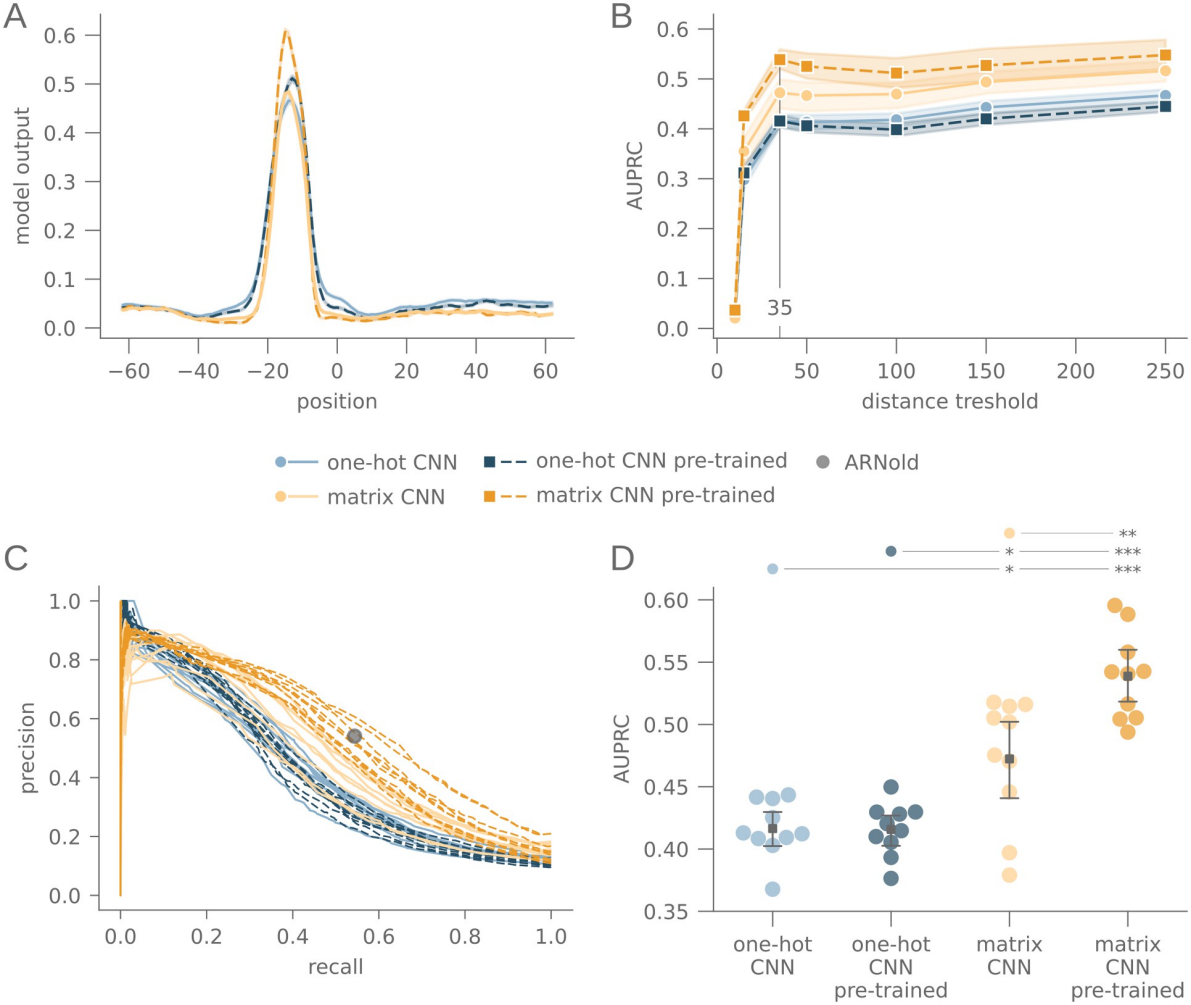
Transcriptome annotation of intrinsic terminators. (A) Average model output of one-hot CNN and matrix CNN with and without pre-training, relative to the position of transcription termination sites identified with SEnd-seq. (B) Average area under precision-recall curve for a transcriptome-wide search for transcription terminators in *E. coli*. The distance to transcription termination sites identified with SEnd-seq is used as ground truth. The distance threshold, up to which a predicted terminator is attributed to a close-by termination site, is varied and shown on the x-axis. (C) Precision-recall curve for all models at a distance threshold of 35 nt, in comparison to precision and recall of ARNold. (D) Area under precision-recall curve at a distance threshold of 35 nt. The p-value of the Wilcoxon rank-sum test between each model *x* and *y* is indicated as coloured dot above model *x*, and as asterisks above model *y*, with *: *p* ≤ 0.05, **: *p* ≤ 0.005, ***: *p* ≤ 0.001. N = 10 for each model type in A and B.

We predicted terminators around the determined transcript ends with all trained models (Fig 4A). The average model output peaked at up to 35 nt upstream of the termination site for all our model types, where the terminator hairpin is located.

We then scanned the *E. coli* transcriptome for terminators, and calculated precision and recall, using the determined transcript ends as ground truth. Predicted terminators were counted as true positives if the distance to the next SEnd-seq hit did not exceed 10 nt, and false positive otherwise. SEnd-seq hits with no terminator predicted in the 10 closest nucleotides were counted as false negatives. We calculated the area under precision-recall curve (AUPRC) for these results, and compared them with the AUPRC for distance thresholds of 15, 35, 50, 100, 150 and 250 nts (Fig 4B). The AUPRC reached a local maximum at a distance threshold of 35 nt. Considering that the model input spans over 75 nt and the termination site is placed upstream of the detected terminator, a distance of about 35 nt to the termination site is expected for large terminators of a maximum length of 70 nt, while the termination site would still be included in the model input.

We compared the neural network based predictions with ARNold as a reference method. We computed the precision and recall at a distance threshold of 35 nt for ARNold’s 1304 hits and compared them to the precision-recall curves of the neural-networks based transcriptome scans (Fig 4C). The transcriptome-scan of the pre-trained matrix CNN was of the same reliability as the one performed with ARNold. Both matrix CNNs outperformed the two one-hot CNNs, and the pre-training of the matrix CNN significantly improved the result. Thus, the ability of the models to detect terminators in the transcriptome was positively correlated with their ability to recognize RNA structures, indicating that structure recognition is a beneficial ability in the case of terminator detection.

## Discussion

We introduced inverse folding based pre-training for different neural network architectures, and demonstrated its effectiveness for the identification of secondary structure motifs of ncRNAs, specifically in the context of identifying intrinsic transcription terminators and tRNAs.

Our inverse pre-training differs from the transfer learning method proposed by Singh et al. [19] in two ways. First, inverse pre-training does not require large numbers of sequences with reference secondary structure, and secondly, inverse pre-training is intended to be family specific by generating training sequences for one specific family of non-coding RNA.

The effectiveness of pre-training is observed consistently across different neural network architectures, covering different CNNs as well as an LSTM model and both ncRNA families under our investigation. All models were able to learn to detect terminators with considerably high precision, recall and F1-score on the validation set (Fig 3 and S1B–S1D Fig). Additionally, all models were able to detect terminators in a transcriptome-wide search, including previously unseen sequences (Fig 4 and S7 Fig).

The non-pretrained one-hot CNN shows limitations in the capability of recognizing RNA structures. However, with matrix encoding and inverse pre-training we identified two strategies to enhance the structure recognition and to direct the attention of neural networks on secondary structures.

The structural information obtained during pre-training was apparently preserved throughout the main training with real terminator data. We were able to detect the sustainability of this pre-training effect in mutation experiments, in which we detected a higher impact of the stem structure on pre-trained models compared to models without inverse pre-training (Fig 2). Interestingly, the effect was transferable onto another deep learning topology, more specifically an LSTM (S8B and S8C Fig), where the impact of the structure on terminator detection was also enhanced after pre-training the model.

Second, we adjusted the encoding technique to provide more information about possible base pairings in the terminator sequence. We used a matrix to encode G–C, A–U and G–U pairings, with entries according to the pairing stability. By providing information about the stability directly, this feature was moved further into focus of the learning process. Interestingly, models which had an enhanced structure recognition ability also performed better in detecting all terminators in the validation set and thus had a higher precision and recall on the validation set (Fig 3), and the matrix CNN also had a higher precision and recall in the transcriptome scan (Fig 4D). The pre-trained LSTM also showed a slightly higher response towards structural changes of the stem region (S8B and S8C Fig). However, it did not outperform the naive LSTM in precision and recall on the validation set (S1C and S1D Fig) nor in the transcriptome scan (S7D Fig).

It must be noted that all training and validation in the course of this study have been performed with data from the same species, *B. subtilis* and *E. coli*. Transcription terminators in other species might differ in various characteristics, and thus be overlooked when no similar terminators are included in the training data. For example, in genomes with a divergent GC-content, terminators could be expected to have a differing U-frequency in the 3’-end. Nonetheless, the existence of a U-tail is known to be required for the function of a terminator [59]. It is therefore advantageous that the recognition of U-tails is not completely vanished when the models learn RNA structures.

The inevitable restriction to *B. subtilis* and *E. coli* data also inherently limits the generalizability of the trained models. Recently, [59] re-tested intrinsic terminators from the study of [58] on *B. subtilis*. They did not find sufficient termination efficiency for 5 of the 80 tested sequences. Like all data-driven methods, the models we present here reproduce biases and errors from the underlying data sets. With the increasing availability of high-throughput methods like term-seq [46] and SEnd-seq [48], however, more large-scale studies might be available soon. A systematic comparison with such experimental data will provide a clear characterization of those terminators that could be identified by our deep learning approach, but not by previous approaches (Fig 4C).

## Conclusion

Deep learning models for RNA secondary structure lack precision whenever the structure of a target RNA is not represented or underrepresented in the training data. Our newly proposed inverse folding based pre-training method promises to overcome this limitation whenever the target secondary structure is sufficiently well understood. Our pre-training easily extends to larger and more complex RNA structures, and provides an almost unlimited number of samples for the pre-training rounds. We successfully tested RNAinverse to generate pre-training data [64, 65]. For structure classifications with even fewer known RNA structures or structural patterns defined by more abstract RNA shapes, the method could be further expanded by applying other inverse folding algorithms [63]. As we demonstrated, inverse pre-training does not hinder the model from learning additional features in the subsequent main training, while the features learned during the pre-training do not fall into oblivion.

RNA secondary structure depends not only on the primary sequence, but also on other factors such as RNA modifications [66] which are known to be identifiable by neural networks [67, 68]. While it is difficult to combine such factors with secondary structure constraints in model-based approaches, neural networks provide an attractive model-free alternative. From this perspective, our inverse folding based pre-training provides means by which model-based understanding can be transferred to a neural network. Identifying signals of less understood factors of an ncRNA family is then left to the subsequent main training of the network. By investigating the structure prediction of the stem loop and the motif recognition of the U-tail, our work constitutes a first step in this direction.

## Supporting information

S1 Fig Model architecture and measure of LSTM models. Model architecture (A), F1-score (B), area under precision-recall curve (C) and precision-recall curve (D) of the LSTM with and without pre-training on the validation data.
(TIF)

S2 Fig Wilcoxon rank-sum test. P-values of Wilcoxon rank-sum tests of the F1-score (Figs 3A and S1A), the area under precision-recall curve tested on the validation data sets (Fig 2B and S1B Fig), and the area under precision-recall curve tested on the transcriptome scan (Figs 4D and S7D).
(TIF)

S3 Fig GC–content of training and pre-training data. GC–content of terminators and negative data in the training set as well as the inverse-folding based data and negative data in the pre-training set.
(TIF)

S4 Fig Impact of section mutations in terminators. Relative activation impact of pre-trained and non-pre-trained one-hot CNN (A, D), matrix CNN (B, E) and one-hot LSTM (C, F), as well as relative detection impact of ARNold (G), for all *k* = 10 validation sets, corresponding to point mutations in different terminator sections. For *k* = 1, …, 10 and *n* ∈ {93, 84, 102, 91, 94, 92, 93, 113, 99, 92}.
(TIF)

S5 Fig Impact of base pair mutations in terminators. Model output of pre-trained and non-pre-trained one-hot CNN (A, D), matrix CNN (B, E) and one-hot LSTM (C, F), as well as detection rate of ARNold (G), corresponding to an increased number of mutated base pairs in terminators. The mutations either retain (blue) or disrupt (red) the pairing in the stem. The model output is averaged over *k* = 10 trained models.
(TIF)

S6 Fig Impact of base pair mutations in tRNAs. Model output of pre-trained and non-pre-trained one-hot CNN (A, D), matrix CNN (B, E) and one-hot LSTM (C, F), corresponding to an increased number of mutated base pairs in tRNAs. The mutations either retain (blue) or disrupt (red) the pairing in the stem. The model output is averaged over *k* = 10 trained models.
(TIF)

S7 Fig Transcriptome annotation of intrinsic terminators with LSTM models. (A) Average model output of the LSTMs with and without pre-training, relative to the position of transcription termination sites identified with SEnd-seq. (B) Average area under precision-recall curve for a transcriptome-wide search for transcription terminators in *E. coli*. The distance to transcription termination sites identified with SEnd-seq is used as ground truth. The distance threshold, up to which a predicted terminator is attributed to a close-by termination site, is varied and shown on the x-axis. (C) Precision-recall curve for LSTMs with and without pre-training at a distance thresholds of 35 nt, in comparison to precision and recall of ARNold. (D) Area under precision-recall curve at a distance threshold of 35 nt. The p-value of the Wilcoxon rank-sum test between each model *x* and *y* is indicated as coloured dot above model *x*, and as asterisks above model *y*, with **: *p* ≤ 0.005. N = 10 for each model type in A and B.
(TIF)

S8 Fig Impact of sequence and structure on terminator and tRNA recognition of LSTM models. (A) Relative activation impact of terminator sections on LSTM models. Random mutations were introduced in each of the 7 sections of the transcription terminators. The relative activation impact on the models is calculated from the difference between the model output corresponding to the original sequences and sequences with random nucleotide mutations in half of all nucleotides per section. (B) Relative activation impact of the base pairings in the stem of terminators on LSTM models, for a growing number of mutated base pairs. The relative activation impact is calculated from the difference between the model output corresponding to mutations which retain or disrupt the pairing state in the stem structure. (C) Relative activation impact of the base pairings in the stems of tRNAs on LSTM models, for a growing number of mutated base pairs. The relative activation impact is calculated from the difference between the model output corresponding to mutations which retain or disrupt the pairing state in the stem structure. (A), (B): For *k* = 1, …, 10 and *n* ∈ {93, 84, 102, 91, 94, 92, 93, 113, 99, 92} (C): For *k* = 1, …, 10 and *n* ∈ {198, 203, 194, 202, 201, 201, 199, 201, 194, 201}.
(TIF)
