# Supplementary figures and images for: Inverse folding based pre-training for the reliable identification of intrinsic transcription terminators
Source: PLoS Comput Biol. 2022 Jul 7;18(7):e1010240. doi: 10.1371/journal.pcbi.1010240 (PMC9262186; doi:10.1371/journal.pcbi.1010240)

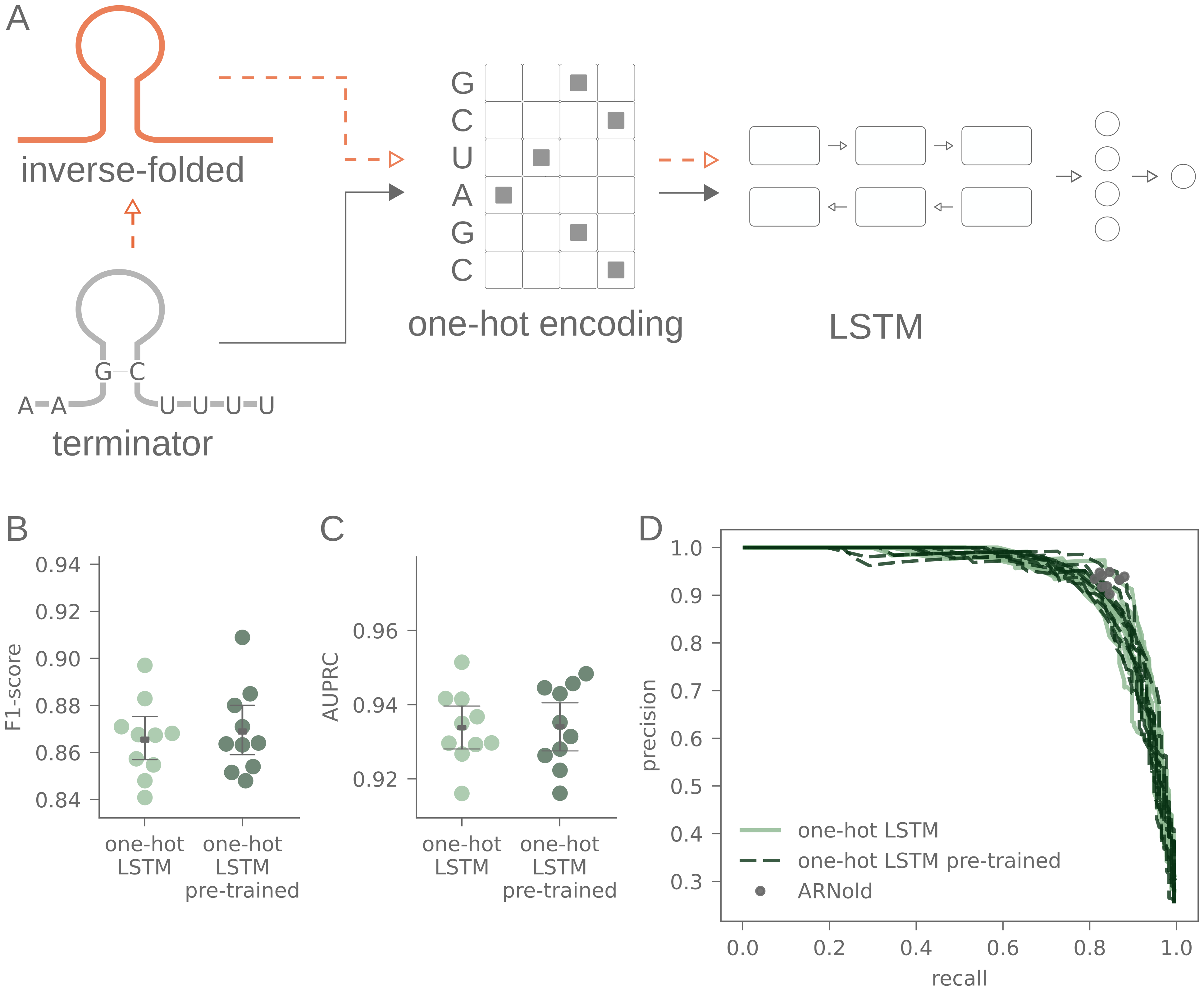

Supplement: S1 Fig — Model architecture (A), F1-score (B), area under precision-recall curve (C) and precision-recall curve (D) of the LSTM with and without pre-training on the validation data. (TIF) [file pcbi.1010240.s001.tif]

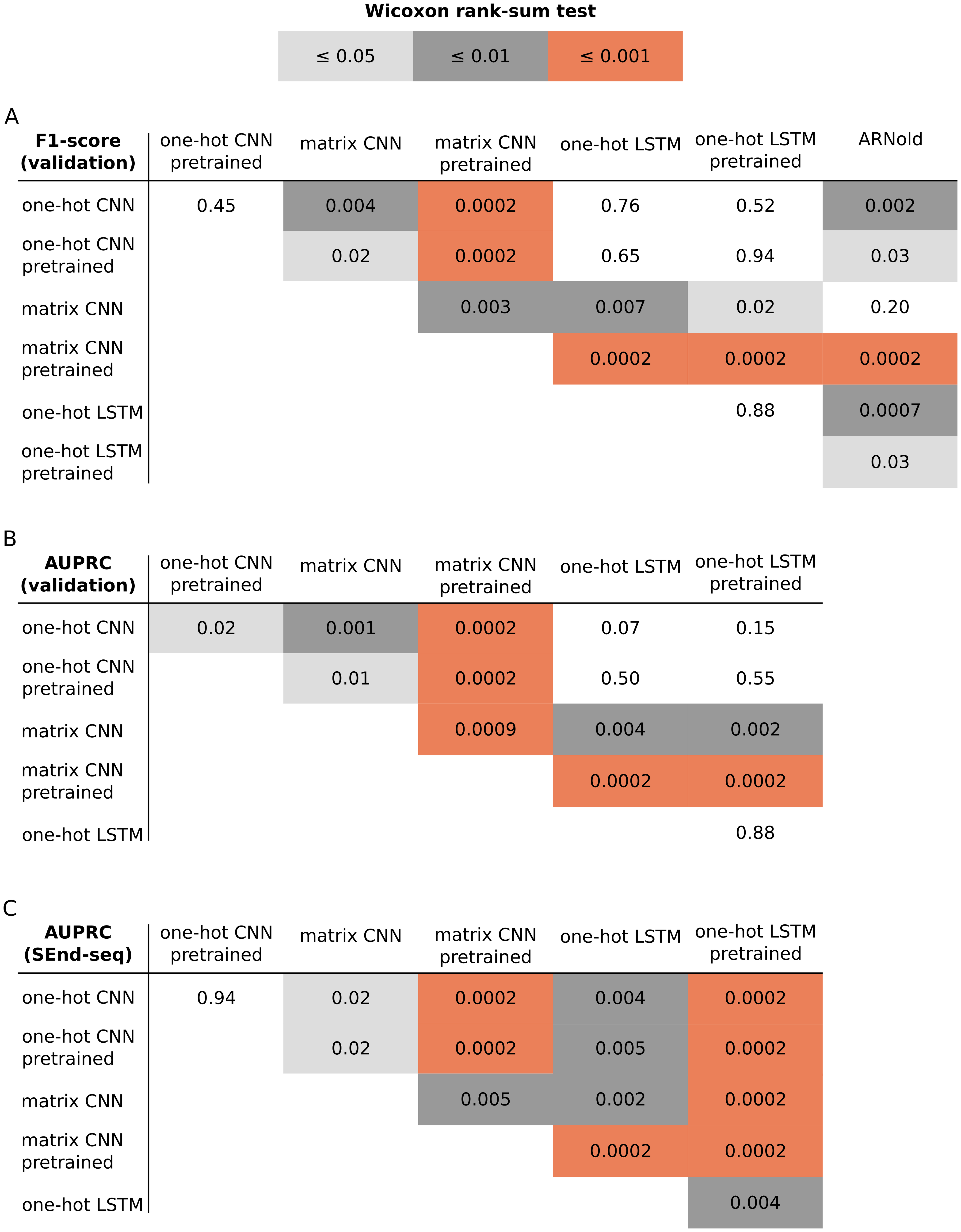

Supplement: S2 Fig — P-values of Wilcoxon rank-sum tests of the F1-score (Figs 3A and S1A), the area under precision-recall curve tested on the validation data sets (Fig 2B and S1B Fig), and the area under precision-recall curve tested on the transcriptome scan (Figs 4D and S7D). (TIF) [file pcbi.1010240.s002.tif]

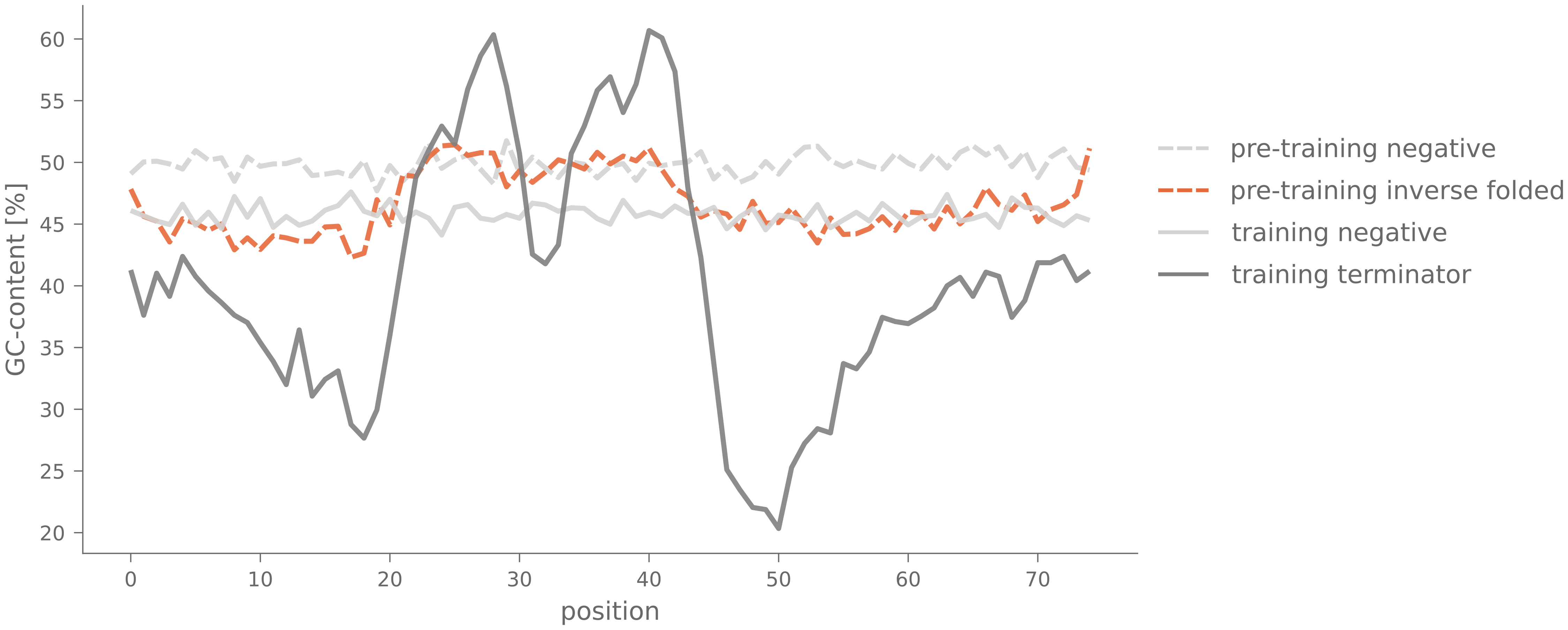

Supplement: S3 Fig — GC–content of terminators and negative data in the training set as well as the inverse-folding based data and negative data in the pre-training set. (TIF) [file pcbi.1010240.s003.tif]

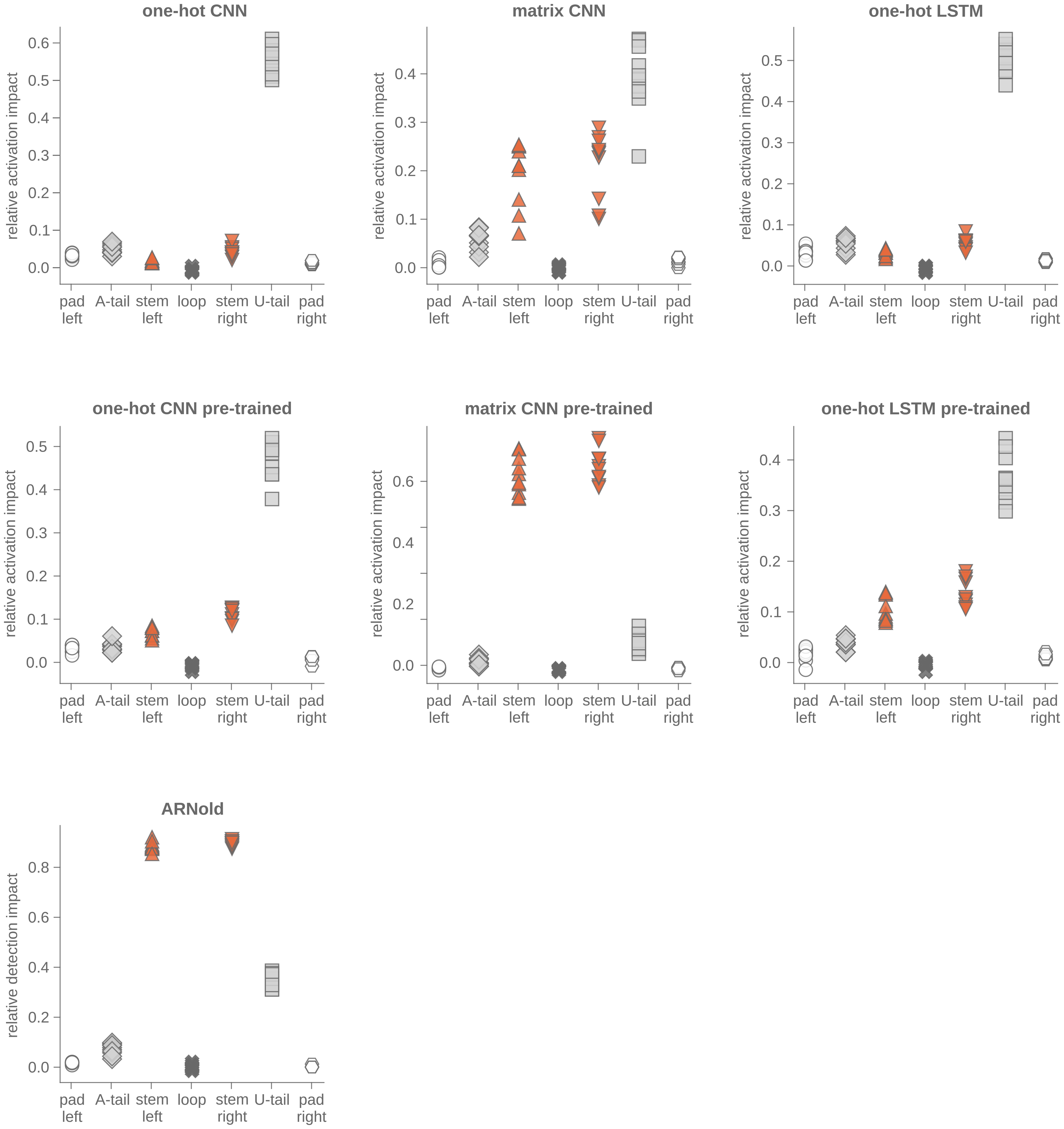

Supplement: S4 Fig — Relative activation impact of pre-trained and non-pre-trained one-hot CNN (A, D), matrix CNN (B, E) and one-hot LSTM (C, F), as well as relative detection impact of ARNold (G), for all k = 10 validation sets, corresponding to point mutations in different terminator sections. For k = 1, …, 10 and n ∈ {93, 84, 102, 91, 94, 92, 93, 113, 99, 92}. (TIF) [file pcbi.1010240.s004.tif]

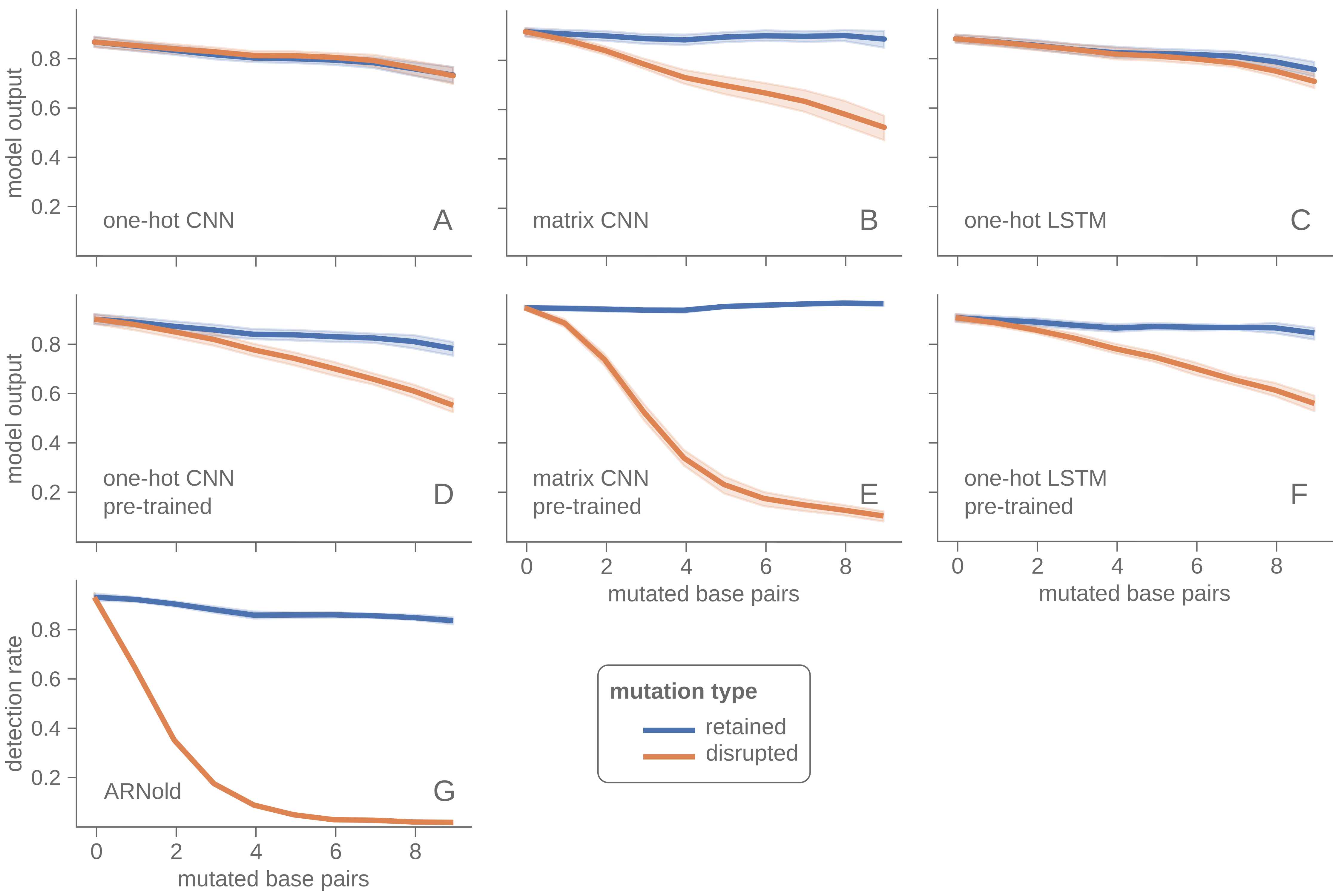

Supplement: S5 Fig — Model output of pre-trained and non-pre-trained one-hot CNN (A, D), matrix CNN (B, E) and one-hot LSTM (C, F), as well as detection rate of ARNold (G), corresponding to an increased number of mutated base pairs in terminators. The mutations either retain (blue) or disrupt (red) the pairing in the stem. The model output is averaged over k = 10 trained models. (TIF) [file pcbi.1010240.s005.tif]

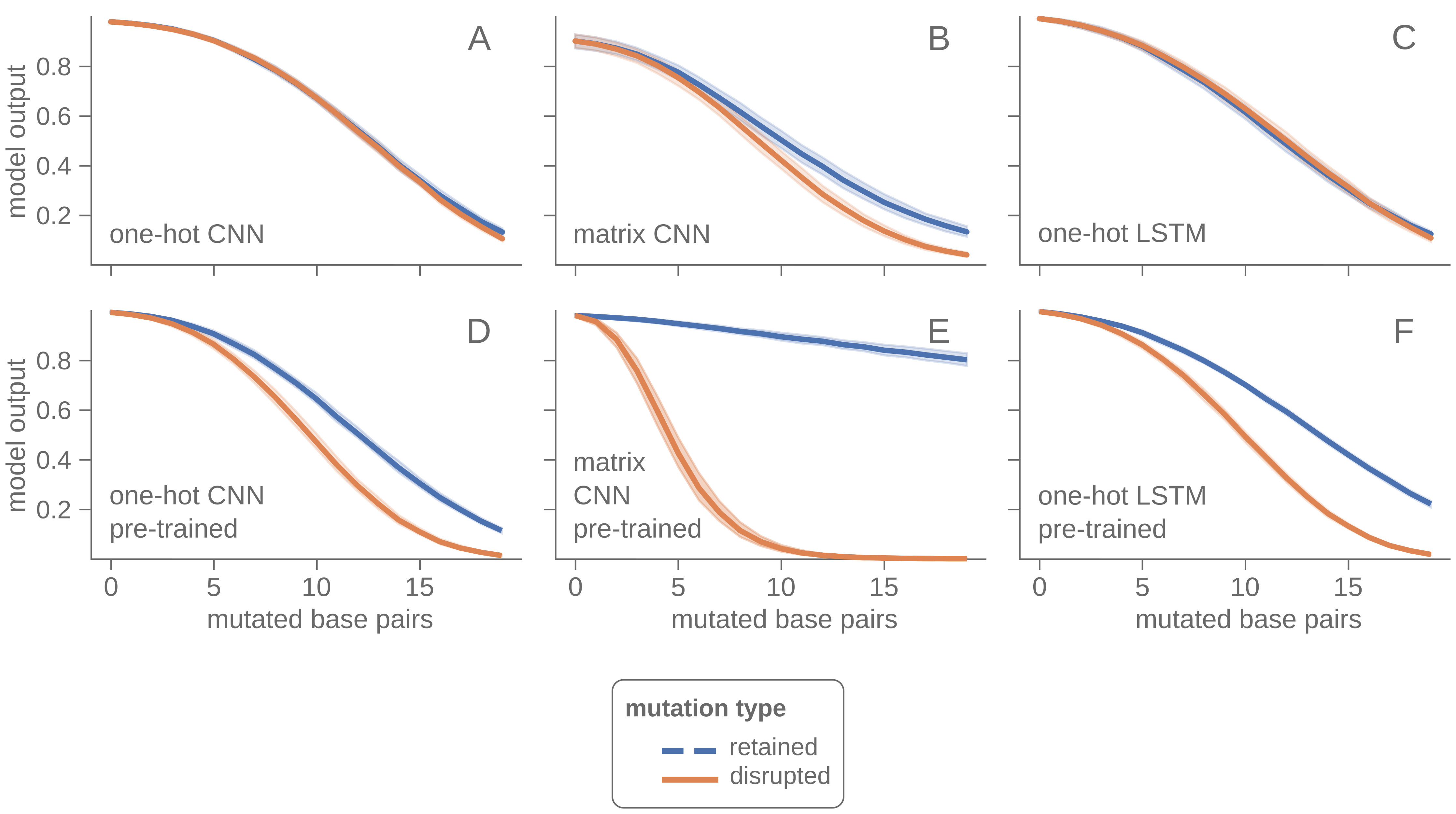

Supplement: S6 Fig — Model output of pre-trained and non-pre-trained one-hot CNN (A, D), matrix CNN (B, E) and one-hot LSTM (C, F), corresponding to an increased number of mutated base pairs in tRNAs. The mutations either retain (blue) or disrupt (red) the pairing in the stem. The model output is averaged over k = 10 trained models. (TIF) [file pcbi.1010240.s006.tif]

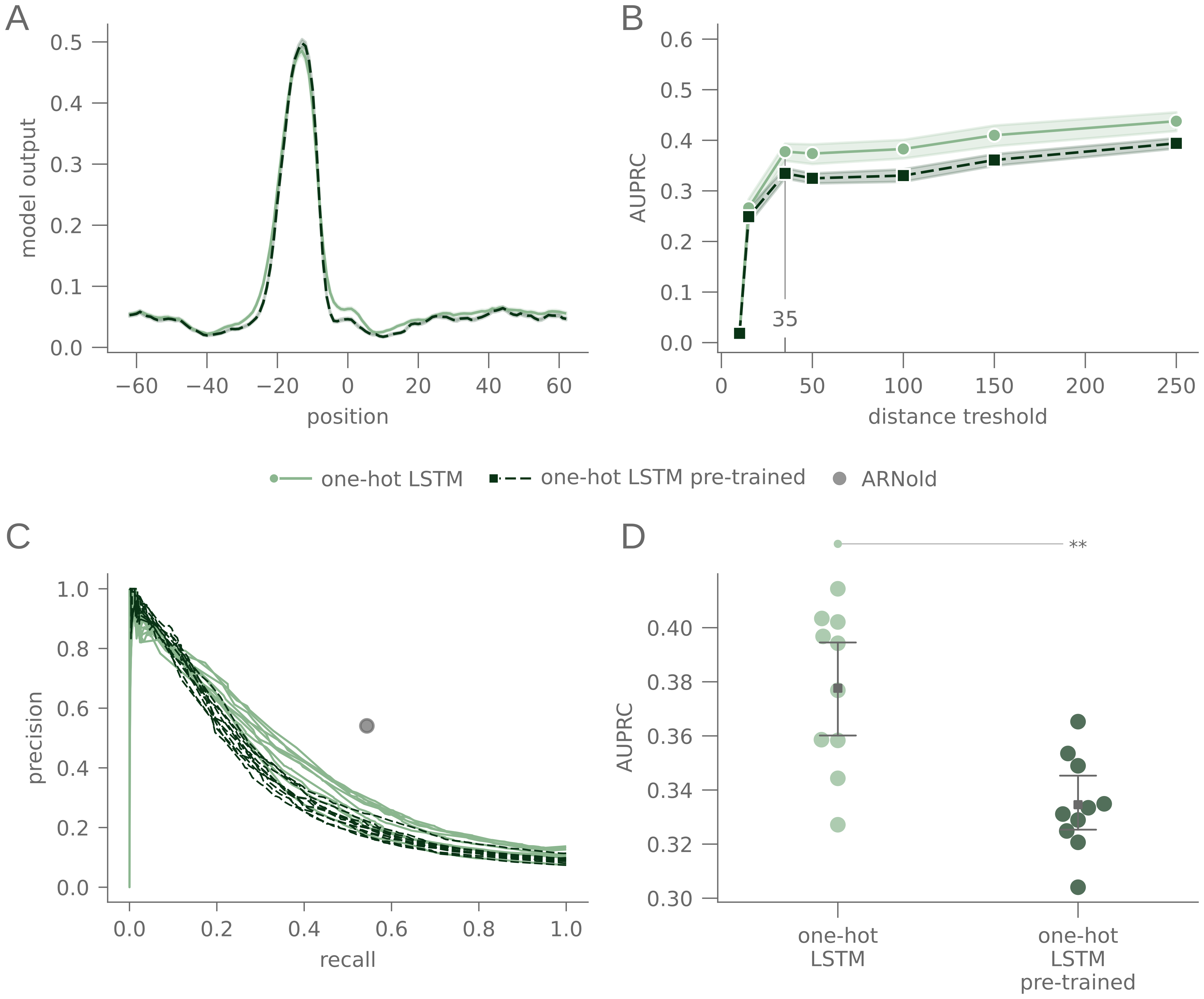

Supplement: S7 Fig — (A) Average model output of the LSTMs with and without pre-training, relative to the position of transcription termination sites identified with SEnd-seq. (B) Average area under precision-recall curve for a transcriptome-wide search for transcription terminators in E. coli. The distance to transcription termination sites identified with SEnd-seq is used as ground truth. The distance threshold, up to which a predicted terminator is attributed to a close-by termination site, is varied and shown on the x-axis. (C) Precision-recall curve for LSTMs with and without pre-training at a distance thresholds of 35 nt, in comparison to precision and recall of ARNold. (D) Area under precision-recall curve at a distance threshold of 35 nt. The p-value of the Wilcoxon rank-sum test between each model x and y is indicated as coloured dot above model x, and as asterisks above model y, with **: p ≤ 0.005. N = 10 for each model type in A and B. (TIF) [file pcbi.1010240.s007.tif]

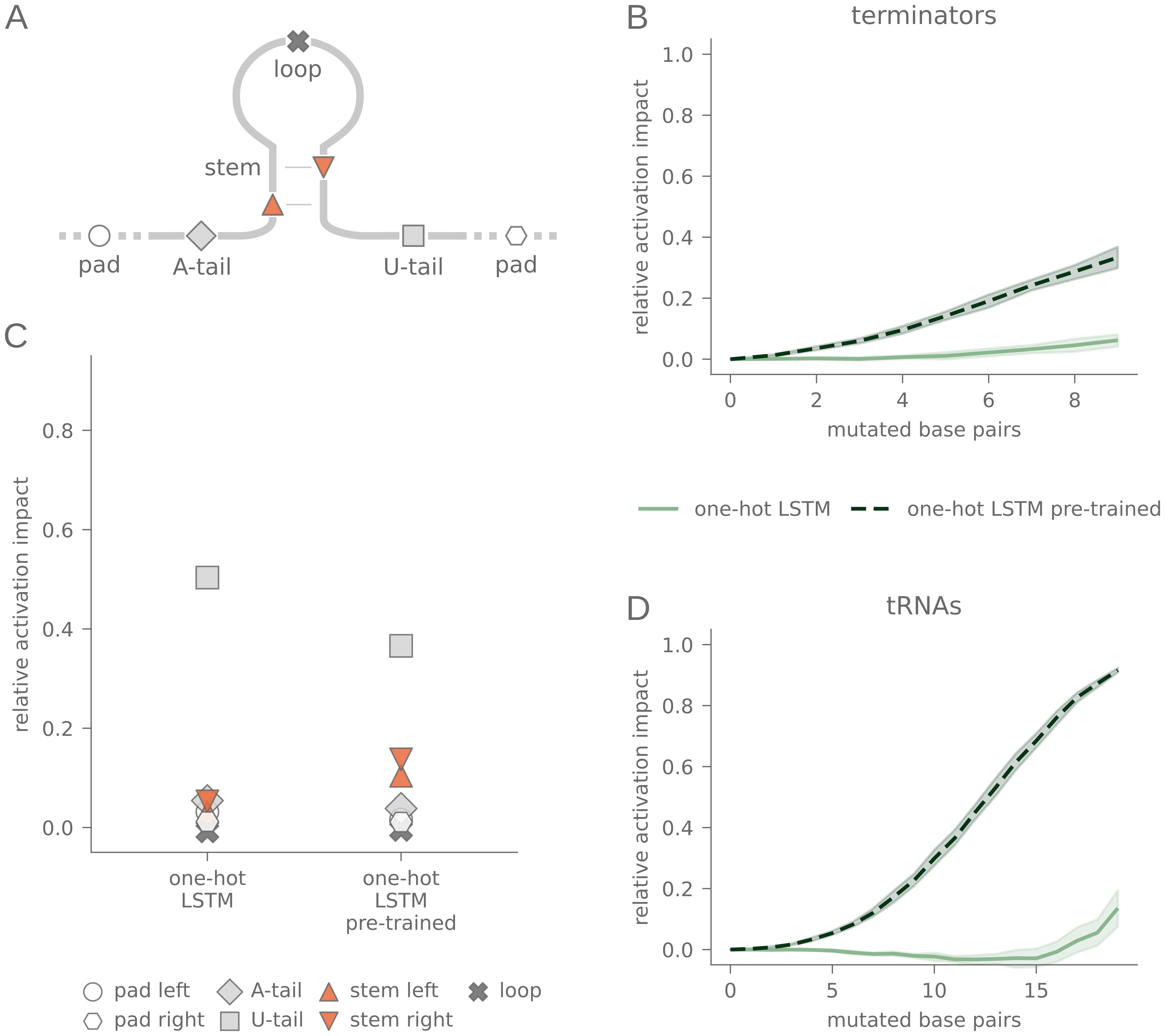

Supplement: S8 Fig — (A) Relative activation impact of terminator sections on LSTM models. Random mutations were introduced in each of the 7 sections of the transcription terminators. The relative activation impact on the models is calculated from the difference between the model output corresponding to the original sequences and sequences with random nucleotide mutations in half of all nucleotides per section. (B) Relative activation impact of the base pairings in the stem of terminators on LSTM models, for a growing number of mutated base pairs. The relative activation impact is calculated from the difference between the model output corresponding to mutations which retain or disrupt the pairing state in the stem structure. (C) Relative activation impact of the base pairings in the stems of tRNAs on LSTM models, for a growing number of mutated base pairs. The relative activation impact is calculated from the difference between the model output corresponding to mutations which retain or disrupt the pairing state in the stem structure. (A), (B): For k = 1, …, 10 and n ∈ {93, 84, 102, 91, 94, 92, 93, 113, 99, 92} (C): For k = 1, …, 10 and n ∈ {198, 203, 194, 202, 201, 201, 199, 201, 194, 201}. (TIF) [file pcbi.1010240.s008.tif]
